# Functional MRI of Challenging Food Choices: Forced Choice between Equally Liked High- and Low-Calorie Foods in the Absence of Hunger

**DOI:** 10.1371/journal.pone.0131727

**Published:** 2015-07-13

**Authors:** Lisette Charbonnier, Laura N. van der Laan, Max A. Viergever, Paul A. M. Smeets

**Affiliations:** 1 Image Sciences Institute, University Medical Center Utrecht, Utrecht, The Netherlands; 2 Division of Human Nutrition, Wageningen University, Wageningen, The Netherlands; University of Colorado Medical School, UNITED STATES

## Abstract

We are continuously exposed to food and during the day we make many food choices. These choices play an important role in the regulation of food intake and thereby in weight management. Therefore, it is important to obtain more insight into the mechanisms that underlie these choices. While several food choice functional MRI (fMRI) studies have been conducted, the effect of energy content on neural responses during food choice has, to our knowledge, not been investigated before. Our objective was to examine brain responses during food choices between equally liked high- and low-calorie foods in the absence of hunger. During a 10-min fMRI scan 19 normal weight volunteers performed a forced-choice task. Food pairs were matched on individual liking but differed in perceived and actual caloric content (high-low). Food choice compared with non-food choice elicited stronger unilateral activation in the left insula, superior temporal sulcus, posterior cingulate gyrus and (pre)cuneus. This suggests that the food stimuli were more salient despite subject’s low motivation to eat. The right superior temporal sulcus (STS) was the only region that exhibited greater activation for high versus low calorie food choices between foods matched on liking. Together with previous studies, this suggests that STS activation during food evaluation and choice may reflect the food’s biological relevance independent of food preference. This novel finding warrants further research into the effects of hunger state and weight status on STS, which may provide a marker of biological relevance.

## Introduction

We are continuously exposed to food and during the day we make many choices regarding food consumption. As the prevalence of overweight and obesity continues to rise [1], research on food choice is becoming of increased interest because food choices play an important role in determining energy intake. Normal human physiology is innately geared towards obtaining food, which is a powerful reinforcer [2]. Easy availability of tasty foods has caused a shift from eating for survival to eating for the pleasure obtained from food reward (hedonic eating) [3–5]. Consequently, metabolic need no longer governs human eating behavior [3,6].

In the past two decades, functional magnetic resonance imaging (fMRI) has become an established method for investigating food-related brain responses [7]. Especially brain responses to the presentation of food pictures have been widely investigated with fMRI [8–17]. Several of these studies have investigated neural responses to pictures of high and low calorie foods [9,13,15–19]. They have shown that high calorie foods are more rewarding than low calorie foods. However, these studies were limited by studying high versus low calorie food viewing contrast in the absence of a choice context. Furthermore, the food stimuli were not matched on liking, which might explain the difference in reward. In addition, participants were usually in a hungry condition, which increases food reward [18].

In addition, the neuroimaging literature on decision making including the investigation of food choices is growing [16,20–29]. In these studies various manipulations were used to examine different aspects of food choice-related processing in the brain including the effects of taste [25] and willingness to pay for different foods types [26]. However, studies investigating food choice between foods differing in caloric content have, to our knowledge, not been described in the literature. The absence of literature might be explained by the complexity of the topic because of the many factors that may influence the choice between foods differing in caloric content. These factors include the food’s palatability, personality traits and motivational state [30–32]. Hunger increases the rated pleasantness of foods and brain regions involved in reward processing are stronger activated when people are viewing pictures of foods in a hungry state [18]. In line with these findings it is often assumed that there would be minor differences in rewarding properties between high and low calorie food in a sated condition. Yet, studies investigating this are lacking. This is important because it has been shown that many people eat in the absence of hunger. In an environment where food is scarce this is an adaptive characteristic because energy can be stored for later in adipose tissue. However, in our Western society this eventually contributes to overweight [33,34]. To our knowledge, it is unknown which neural mechanisms subserve this phenomenon.

Therefore, the aim of the present study is to investigate the neural mechanisms underlying the choice between equally liked high calorie and low calorie foods in the absence of hunger. We predict minimal differences between brain responses during high versus low calorie food choices, as the subjects are sated and the choices are matched on liking. Because the majority of the food evaluation studies examine the food versus non-food contrasts, we additionally aim to investigate the neural mechanisms underlying food choice versus non-food choice in the absence of hunger. We hypothesize increased activation during food choice in brain regions predominately involved in attention as foods are thought to be more salient than office utensils (i.e. the non-foods used in this study) [35,36] [12,18].

## Materials and Methods

### Ethics statement

The study was approved by the Medical Ethical Committee of the University Medical Center Utrecht and participants provided written informed consent.

### Participants

Participants were recruited by distributing flyers and posters in the University Medical Center Utrecht and at the university campus. Forty-two participants enrolled in the study. We included healthy participants with a normal weight (i.e., BMI 18–25 kg/m^2^), between 20–40 years old, right-handed, non-smoking, with a stable weight (did not gain or lose > 5 kg in the past 6 months), no use of medication (except aspirin/paracetamol and oral contraceptives) and no current alcohol consumption of > 28 units per week. We excluded participants who scored above average on restraint eating (restraint eating subscale score of the DEBQ could not exceed 2.89 for males and 3.39 for females)[37], since this characteristic is known to influence food relationships [38]. Furthermore, common fMRI exclusion criteria (e.g. claustrophobia, pregnancy and metal implants in the body) and criteria that might influence response to food cues (e.g. food allergies, special diets, eating disorders, gastrointestinal disorders or metabolic or endocrine disease) were used. In addition, runs with any single movement greater than 4 mm translation or 4 degrees rotation were excluded. From the original sample (N = 42), subjects meeting one of the following criteria were excluded for the current analysis: nausea (self-report >5 on a 9-point Likert scale) after test meal consumption (N = 8), too much hunger (self-report >5 on a 9-point Likert scale) after test meal consumption (N = 1) or prior to the scan (N = 4) and <10 high and low calorie choices during the forced choice fMRI task (N = 10). No subjects had to be excluded for excessive movement (See S1 Table for more details). The 19 remaining participants (9 males, 10 females; age (Mean, SD) = 25.4 ± 5.1; BMI (Mean, SD) = 22 ± 1.6; DEBQ dietary restraint (Mean ± SD): males = 1.82 ±0.66; females = 2.41 ± 0.49) were examined in this study.

### Experimental design

The study consisted of one MRI scan session conducted in the morning. Subjects were scanned after an overnight fast (≥ 10h) after consumption of an ad libitum test meal (a commercially available drink called Nutridrink from Nutricia, see S2 Table for more details). They provided hunger and fullness ratings before and after test meal consumption. These served to ensure that their hunger decreased after test meal consumption. The amount of protein shake consumed ranged from 117–631 ml (Mean, SD = 449.7 ± 170.9 ml). Hunger ratings decreased and fullness ratings increased significantly after protein shake consumption (9-point Likert scale measurements: pre-meal hunger (Mean, SD) = 6.1±1.8; after meal hunger (Mean, SD) = 2.1 ± 1.1; and pre-meal fullness (Mean, SD) = 2.5±1.2; after meal fullness (Mean, SD) = 7.2 ± 1.5).

**Fig 1:**
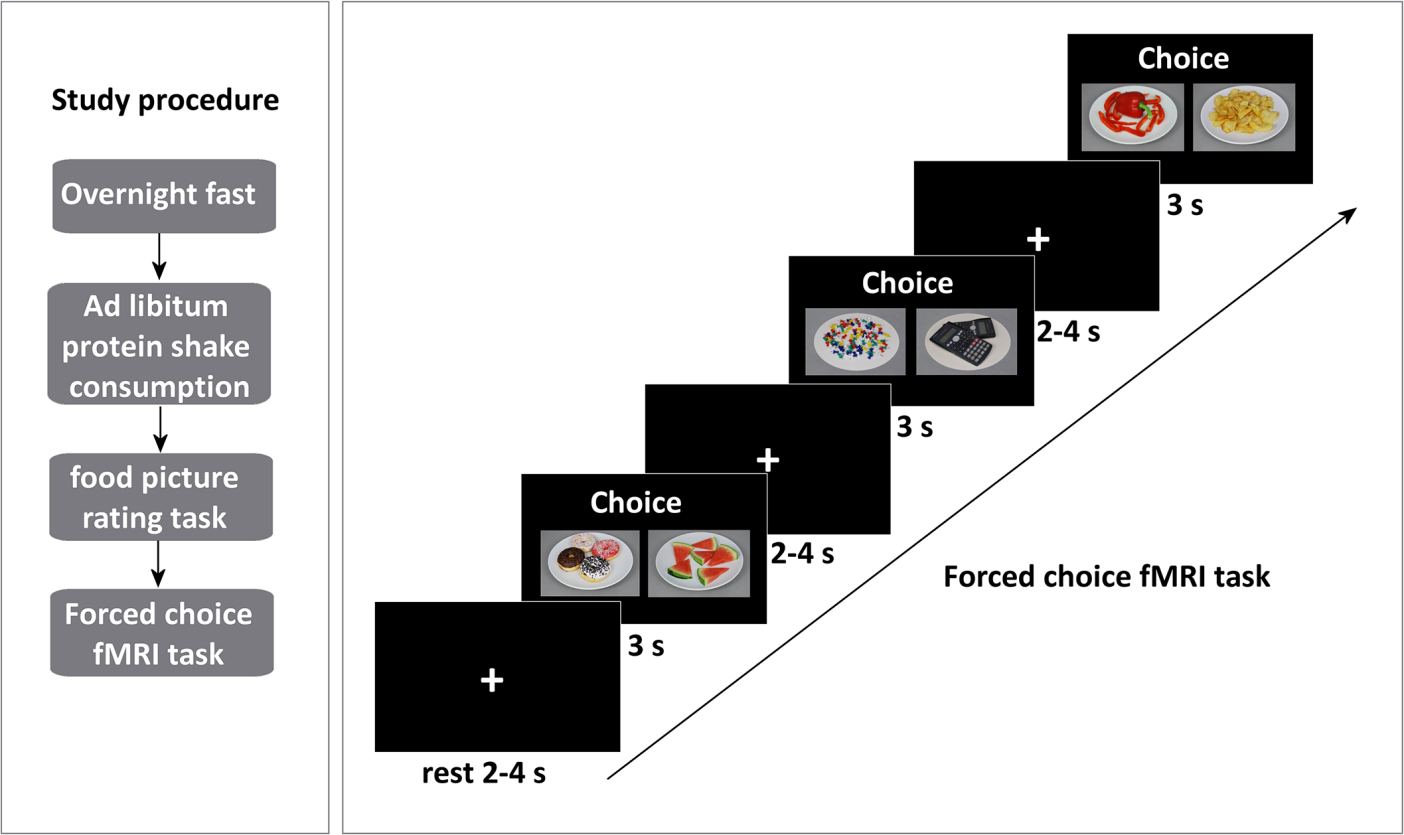
Study procedure & forced choice fMRI paradigm.

Before the scan, participants conducted a computerized food picture rating task (based on [31]). Subsequently, the participants underwent a 30-min MRI scan session. The first functional run consisted of a food and non-food viewing task, the second consisted of a forced choice task. In this paper we report the results of the forced choice task (see Fig 1).

### Tasks

#### Stimuli

The stimuli used in this study were 96 food and 51 non-food images custom-made for this study. The food image set contained mostly snacks, ready for consumption, without package and brand information. The foods can be divided into two categories: high calorie and low calorie images (including both sweet and savoury items). Each food was presented on a plate, filled with the food. The plates were shown on a standardized background. To ensure the background was identical for every picture, each plate was registered to a standardized background with the use of MeVisLab (MeVis Medical Solutions AG, Bremen, Germany) and Elastix [39]. The non-foods were office utensils, depicted in a similar way as the food items, on a white round piece of paper instead of a plate.

#### Food picture rating task

Shortly before the MRI scan the participants executed a food picture rating task which was based on the Leeds Food Preference Questionnaire (LFPQ) [31]. During this task participants rated 96 food pictures on liking, caloric content and healthiness on a 9-point Likert scale. The food pictures were divided in high calorie and low calorie categories (including both sweet and savoury items). All images are freely available on request, see S1 PDF for an overview of all images used in this study. Each food picture was shown for 3 seconds (which was similar to the time the participants had to choose during the forced choice fMRI task). After that the following questions were asked: ‘How much do you like the product?’ (1 not at all—9 very much), ‘How many calories do you think this product consists of?’ (1 very few calories—9 many calories) and ‘How healthy do you think this product is?’ (1 not healthy at all—9 very healthy). The participants received the following instruction: ‘Try to answer the questions as quickly as possible. There are no correct or incorrect answers, it's about your opinion. Don't think too long about an answer, the first answer that occurs to you is usually the best one’.

#### Forced choice fMRI task

**Fig 2:**
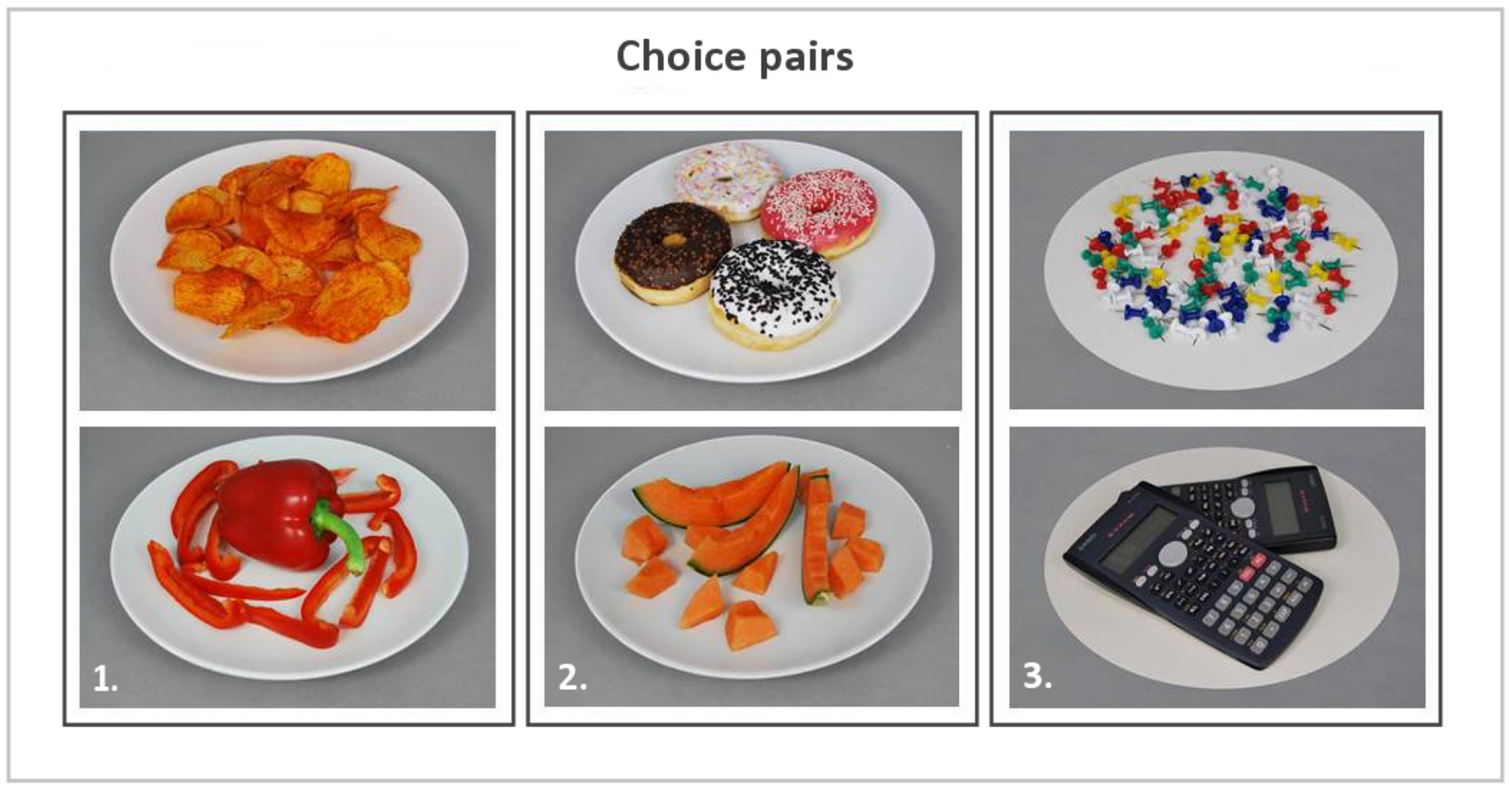
Example of choice pairs. 1: High & low calorie food pair, savoury taste; 2: High & low calorie food pair, sweet taste; 3: Non-food pair.

Based on the ratings collected during the food picture rating task, food pairs were created for each subject. Food pairs were matched on liking (i.e. equal ratings or plus/minus 1 on a 9-point scale) and taste (i.e. sweet or savoury), to make the pairs as equal as possible, but differed in caloric content (i.e. a minimum of 2 points difference on a 9-point scale) (see Fig 2). Each pair was unique although a picture could appear in several food pairs (repetition (means, SD) = 1.17 ± 0.08; range = 1–2). To check whether our manipulations were successful, mean actual caloric content (kcal), perceived caloric content (9-point Likert scale), healthiness (9-point Likert scale) and mean liking (9-point Likert scale) were calculated. As expected, all variables except liking, differed significantly between the choice options within a food choice pair (Table 1). Hence, the study manipulations were effective. The participants were verbally giving the following instructions:”choose the product of which you most want to eat at this moment”, whenever a food pair appeared, and “choose one of the products”, when a non-food pair appeared (without giving any direction or further instructions). In addition to the verbal instruction, each question was shown above every choice pair. Subjects had 3 seconds to indicate their choice. Whenever a subject failed to make a choice within the restricted time, the event was labelled as a missed choice. The choice pairs were projected on a screen with a projector. The subjects viewed the images via a mirror attached to the headcoil. The stimuli were presented in the scanner by using the PRESENTATION software (Neurobehavioral Systems Inc., Albany, CA).The mean ± SD scan duration of the forced choice task was 508 ± 30s. The length of this scan varied between participants due to the variable number of food pairs (Mean ± SD = 40.7 ± 4.8; range = 28–49 food pairs) that could be created per individual. Furthermore, food choices were alternated with non-food choices (i.e., choices between office utensils) to serve as a control condition and to avoid adaptation to the food stimuli. After each choice a fixation cross of variable length (2–4 s), was shown.

**Table 1:** High and low calorie choice options.

	High calorie pictures		Low calorie pictures	
	(ᴍ±*σ*)	range	(ᴍ±*σ*)	range
Actual cal. ^1^	376.2 ± 13	358.4–412	151 ± 26.1**	99.9–193.5
Perceived cal.^2^	7.5 ± 0.6	6.3–8.4	3.1 ± 0.6**	2.1–4.6
Liking^2^	6.7 ± 0.7	5.3–7.5	6.7 ± 0.6 ^ns^	5.4–7.5
Health^2^	3.0 ± 0.7	2–4.6	7.1 ± 0.4 **	6.3–7.9

** Differences between high& low calorie pictures were significant p < 0.001

^ns^ Differences between high& low calorie pictures were not significant

^1^Actual caloric content kcal per 100 grams

^2^ 9-point Likert scale.

### Image acquisition and preprocessing

Scans were performed with a 3 Tesla Philips Achieva MRI scanner (Philips Healthcare, Best, The Netherlands) using an 8-channel SENSE head coil. A high resolution anatomical image (T¹-weighted scan) was acquired at 1 x 1 x 1 mm resolution (TR = 8.4 ms, total scan duration = 473 s). Functional scans were acquired with a T²*-weighted gradient-echo 2D-EPI sequence (TR/TE = 1400/23 ms, flip angle = 72.5°, voxel size = 4 x 4 x 4 mm, FOV = 208 × 119.6 x 256 mm, dynamic scan duration = 1400 ms). Six dummy volumes were automatically discarded. The total number of volumes collected varied between participants due to the different number of food choice pairs that could be generated (range: 295–400 volumes). Data processing was performed with the SPM8 software package (Wellcome Department of Imaging Neuroscience, London, United Kingdom, (http://www.fil.ion.ucl.ac.uk/spm/software/spm8/) run with MATLAB R2012a (The MathworksInc, Natick, MA). The functional images were realigned to the first image. Subsequently, the functional images and the anatomical images were coregistered and normalized to MNI space (Montreal Neurological Institute–International Consortium for Brain Mapping). In addition, the functional images were smoothed with a Gaussian kernel of 8 mm full width at half maximum (FWHM). The mean functional images were visually inspected for artefacts. Furthermore, the realignment parameters of all subjects were also examined.”

### Behavioral data analyses

The behavioral data were analyzed with SPSS statistics 19. The self-report ratings on a 9-point Likert scale (i.e. liking, perceived caloric content and perceived healthiness), actual caloric content, the number of high and low calorie choices made and reaction times (RTs) were normally distributed. Differences in liking, perceived caloric content, actual caloric content and healthiness between the high and low calorie choice options, the choices made and RTs were analyzed by using paired t-tests. In addition, the percentage of high and low calorie choices made was examined by using a one-sample t-test.

### fMRI analyses

The following five conditions were modeled: high calorie food choice, low calorie food choice, non-food choice, spare choices and missed choices. Because participants were free to choose, the number of high and low calorie choices was unequal for most participants (range number high calorie choices = 11–26; range number low calorie choices = 11–38). To control for this bias, balanced designs were created by selecting equal number of choices per condition of interest per subject. In addition, the choices used for this analysis were selected based on a minimum of 2 points difference in the individual caloric content ratings on a 9 point Likert scale (to ensure each food pair differed in perceived caloric content). The choices that did not meet this criterion, in addition to spare choices (i.e. rest choices due to equal number of choice selection) and missed choices, were modeled as separate conditions.

#### High calorie vs low calorie choices

For the high (HCC) versus low calorie choice (LCC) analysis equal numbers of high calorie, low calorie and non-food choices were selected (range 11–21 choices per category). On first level (single subject analysis) the high calorie choice and low calorie choice versus baseline and high versus low calorie choice contrasts (i.e. conditions of interest) were created. On second level (group analysis) one sample t-tests were performed to examine the significant brain activation of the group during the contrasts mentioned above. The statistical parametric map generated of the HCC-LCC contrast, was thresholded at p < 0.001 uncorrected for multiple comparisons with a cluster-extent k = 20 [40]. The statistical parametric maps calculated for the single conditions (i.e. HCC and LCC) were thresholded more conservatively (p < 0.05 FWE corrected for multiple comparisons at whole brain level, k = 10) since these conditions were contrasted against rest.

#### Food vs non-food choices

For the food choice versus non-food choice analysis, equal numbers of food choices (containing both high & low calorie choices) and non-food choices were selected (range 19–26 choices per category). On first level (single subject analysis) the food choice versus non-food choice contrast was created. On second level (group analysis) a one sample t-test was performed to establish the brain regions that are differentially activated by food and nonfood choices. The generated statistical parametric map was thresholded at p < 0.001 uncorrected for multiple comparisons, k = 20 [40].

## Results

### Behavioral data

Overall, the participants chose significantly more low calorie than high calorie foods (LCC percentage (Mean, SD) = 57% ± 11.3%; t = 2.75; p = 0.013). Because all liking ratings were included in the creation of the food choice pairs, liked, neutral but also disliked pairs could be present. To check whether the choices made per category did not differ significantly in liking a paired sample t-tests was conducted. We found no significant difference in liking ratings between high and low calorie choices (see Table 2). See for more detailed ratings per subject, S3 Table. Furthermore, the RT’s of the high calorie choices were significantly larger than the RT’s of the low calorie choices (RT HCC (Mean, SD) = 1.6 s ± 0.4 s; RT LCC (Mean, SD) = 1.5 ± 0.3; t = 2.45; p = 0.025).

**Table 2:** High and low calorie choices.

	High calorie choices		Low calorie choices	
	(ᴍ ± *σ*)	range	(ᴍ ± *σ*)	range
N	17.3 ± 4.2	11–26	23.4 ± 6.1*	11–38
Actual cal. ^1^	366.4 ± 34.3	310.1–416.6	155 ± 36.3**	74.6–226
Perceived cal.^2^	7.4 ± 0.5	6.3–8.3	3.1 ± 0.7**	2.2–4.5
Liking^2^	6.7 ± 0.7	5.6–7.8	6.7 ± 0.7 ^ns^	5.3–7.8
Health^2^	3.1 ± 0.7	1.9–4.5	7.1 ± 0.4**	6.5–7.9

*Difference between high & low calorie choices were significant p = 0.011

**p<0.0001

^ns^ Difference between high & low calorie pictures were not significant

^1^ Actual caloric content kcal per 100 grams

^2^ 9-point Likert scale.

### fMRI data

#### High calorie & low calorie choices vs baseline

**Fig 3:**
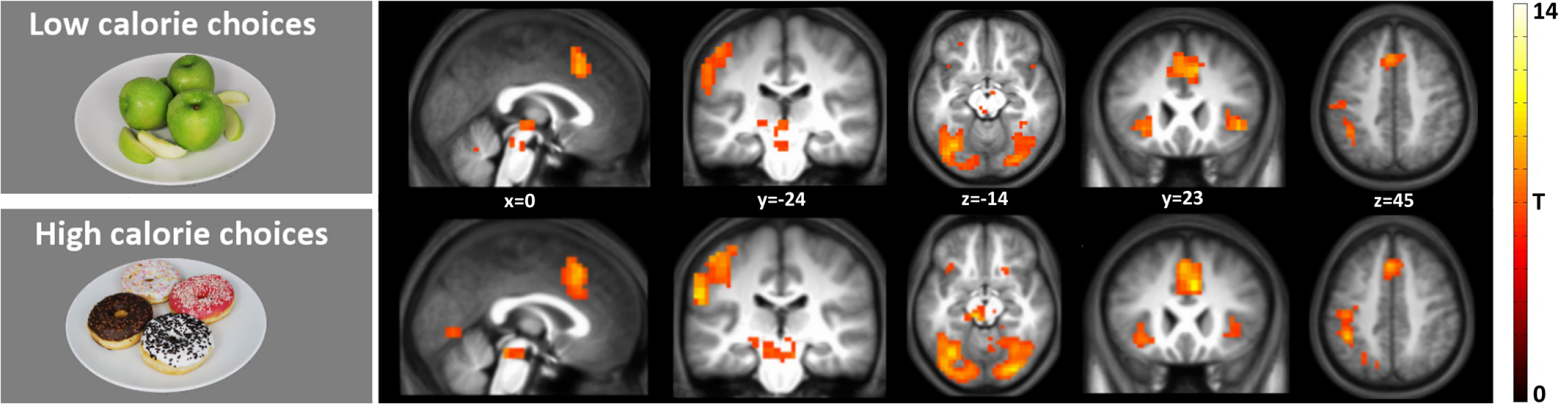
Brain regions with stronger activation in response to HCC and LCC vs baseline. Shown is a T-map thresholded at P<0.05 (FWE-corrected; T>6.25), superimposed on the mean anatomical image of all subjects (MNI-space).

Fig 3 shows the results of the single food contrasts (i.e. high calorie choice and low calorie choice versus baseline) (p < 0.05 FWE corrected, k = 10) are shown. This figure clearly shows the similarity of the brain activation pattern during high and low calorie choice. Regions that were stronger activated compared with rest in both high and low calorie choice include the midbrain, insula, supplemental motor area, middle cingulate gyrus and several visual areas. See S4 and S5 Tables for all the MNI peak coordinates.

#### High calorie vs low calorie choices

**Fig 4:**
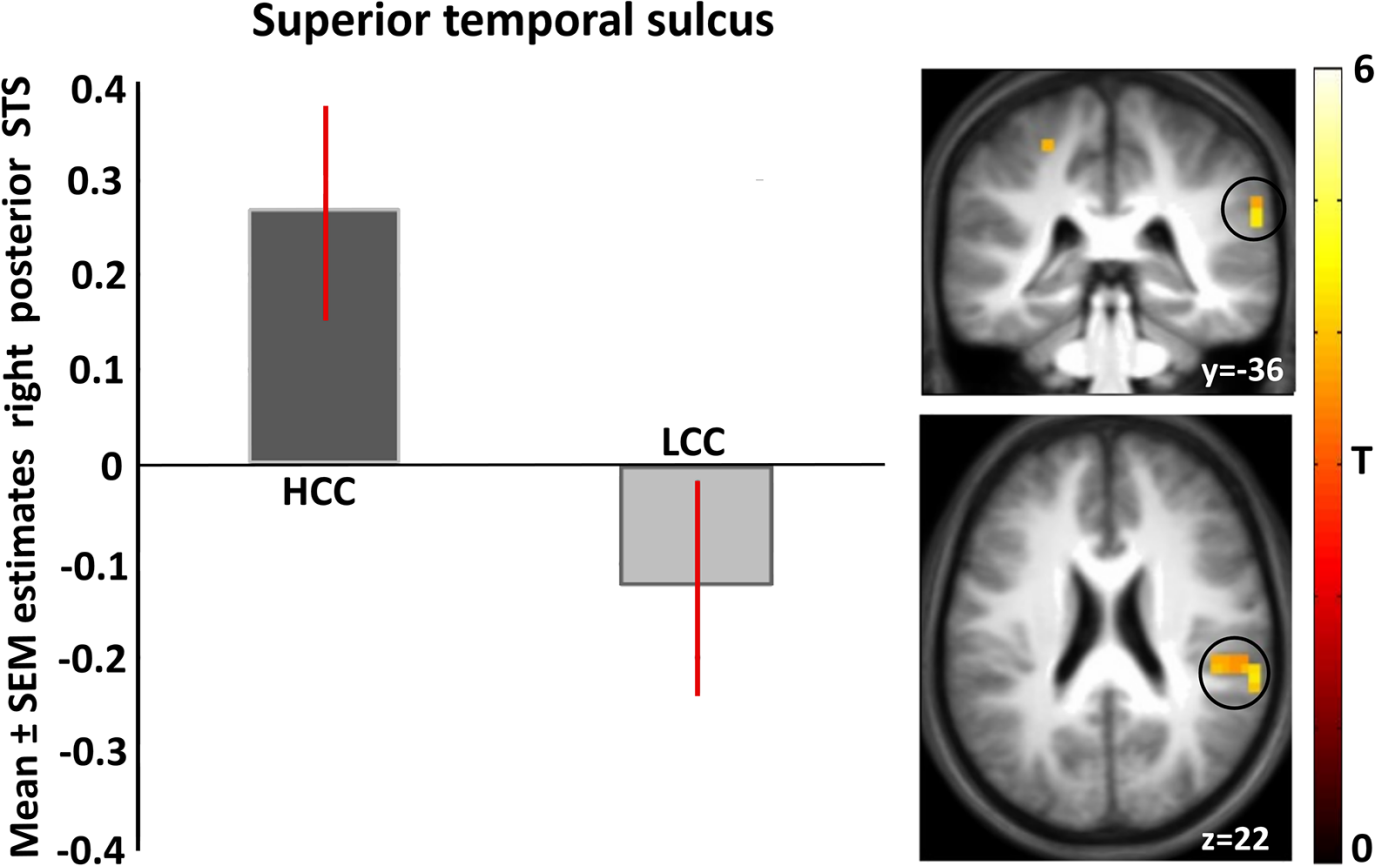
Mean parameter estimates, peak coordinate (62. -36. 22) of the brain region with stronger activation in response to HCC versus LCC. Shown is a T-map for visualization thresholded at T = 3.5 p<0.001 uncorrected for multiple comparisons, superimposed on the mean anatomical image of all subjects (MNI-space).

Few differences were found between high versus low calorie food choices (p<0.001 uncorrected, cluster extent threshold k = 20). Significantly stronger activation was found in the posterior part of the right superior temporal sulcus (See Fig 4; MNI peak coordinate (62, -36, 22); T = 4.32; Z = 3.53) for high versus low calorie choice. This activation did not correlate (pearson r = 0.098, p = 0.691) with the differences in RT’s for the high and low calorie choices (reported in the Behavioural data section). No differences were found in the low compared with high calorie food choice contrast (p<0.001 uncorrected, cluster extent threshold k = 20).

#### Food vs non-food choices

**Fig 5:**
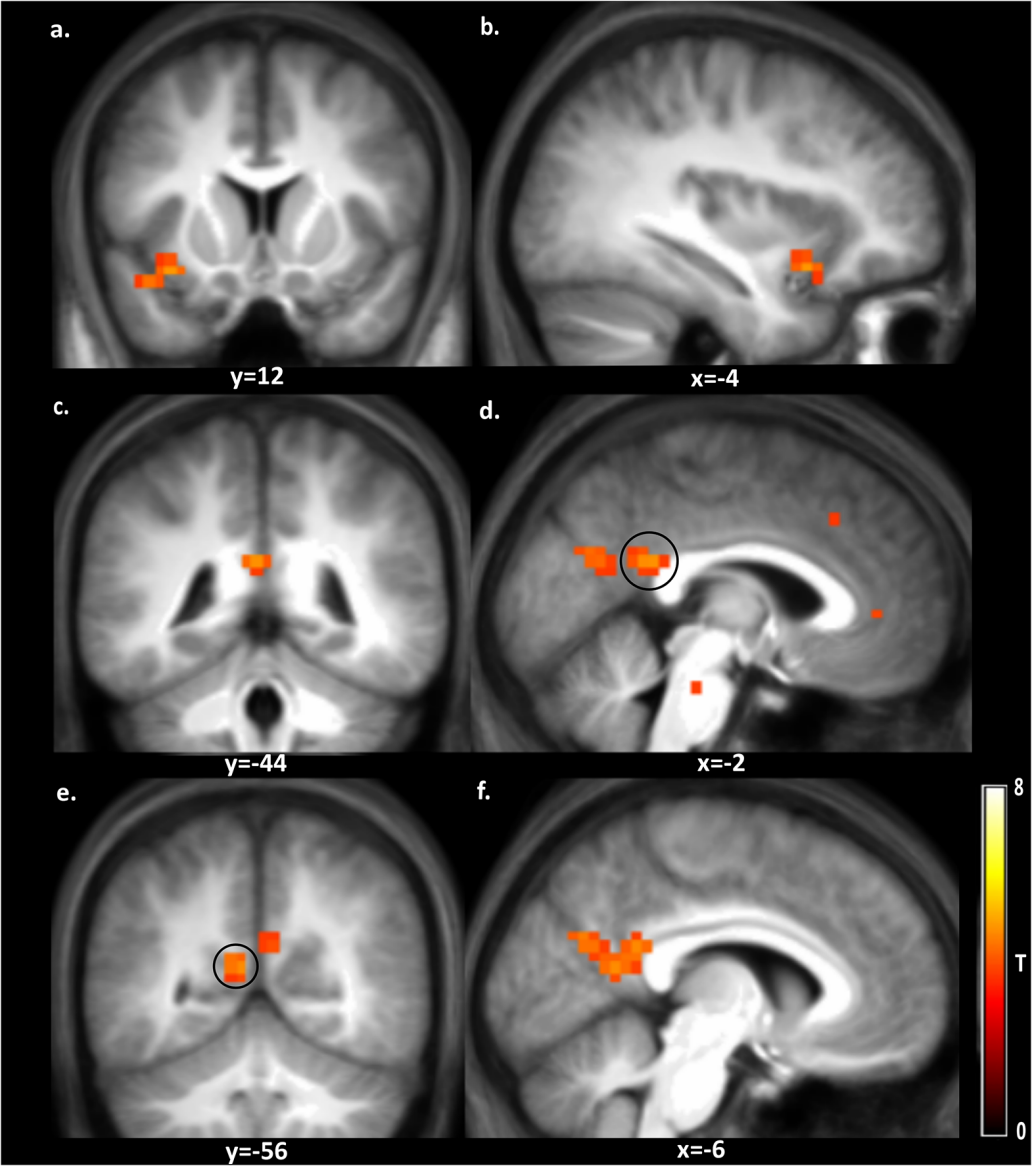
Brain regions with stronger activation in response to food choice versus non-food choice. Shown is a T-map thresholded for visualization purposes at p < 0.001 uncorrected for multiple comparisons (T > 3.6), superimposed on the mean anatomical image of all subjects. a: L, insula and L, superior temporal sulcus; b: L, superior temporal sulcus; c & d: L, posterior cingulate gyrus; e: L, precuneus; f: L, precuneus & L, cuneus; See corresponding peak coordinates in Table 3.

In addition, differences between food and non-food choices in the absence of hunger (p < 0.001 uncorrected, cluster extent threshold k = 20), were investigated. The results are depicted in Fig 5 and the peak coordinates are given in Table 3. Several brain regions, including the insula, posterior cingulate gyrus, cuneus, precuneus and superior temporal gyrus, were stronger activated during food choice (p<0.001 uncorrected, k = 20). In addition the overlap between the food versus non-food choices and the individual contrasts high calorie food choices vs rest and low calorie-food choices vs rest were examined.The left insula was active in all three contrasts (see S1 Fig for more details).

**Table 3:** Brain regions with stronger activation in response to food choices versus non-food choices.

		Peak MNI-coordinates (mm)				
Region	k	x	y	z	T	Z
L, insula (a)	21	-34	12	-14	4.77	3.79
L, superior temporal sulcus (a,b)		-42	12	-18	4.31	3.53
L, posterior cingulate gyrus (c,d)	67	-2	-44	22	4.70	3.75
L, precuneus (e,f)		-6	-56	14	4.64	3.72
L, cuneus (f)		-6	-68	26	4.44	3.60

Peaks are reported for all clusters ≥ 20 voxels at p<0.001 uncorrected for multiple comparisons; L = left and R = right hemisphere; The regions a-f are depicted in Fig 5.

## Discussion

We investigated brain responses during food choices between foods matched on liking but differing in caloric content in the absence of hunger. Although the participants were not above average dietary restraint, not dieting and had a stable weight, they made more low compared to high calorie food choices. In addition, the RTs were higher for the high calorie choices compared to the low calorie choices. We speculate that the subjects in this study choose more low calorie foods because they were in a fed state and were presented with equally liked foods. In this scenario, the low calorie option was, physiologically, the best option to choose to maintain a stable weight.

Brain regions which elicited stronger activation during high and low calorie choice compared to rest include the midbrain, insula, supplemental motor area, middle cingulate gyrus and several regions involved in visual processing. While, the posterior part of the right superior temporal sulcus (STS), was the only region found to be more active during high compared with low calorie choice matched on liking in healthy sated normal-weight volunteers. In addition, this activation did not correlate with the differences in RTs between the high and low calorie choices.

The superior temporal sulcus is thought to be a multifunctional region. The literature on this region is characterized by a large variety of cognitive studies in different fields, ranging from facial recognition to social cognition and theory of mind [41]. Studies investigating face processing have compared brain responses to faces with abstract images having similar contours e.g., Narumoto et al. [42]. In these studies, the right STS is more activated during emotional face expressions (as fear, sadness and happiness). Effects of attention on STS activation have also been reported [43]. Finally, the right posterior STS has been found to be more active during viewing of highly palatable foods versus moderate palatable foods in unrestraint volunteers of normal weight in a fasted state [17]. Although the region that has been reported in Colletta et al., is a different part of the STS than we found (most likely due to the different nature of the task used), it is interesting and suggests that the right posterior STS is not only involved in the processing of faces and emotion but also in other biological relevant processes such as high calorie food evaluation and choice. High calorie food choice is an especially biologically relevant process as these foods are highly energy-dense. Interestingly, the difference in right posterior STS activation seems to be independent of state and palatability since its activation was found in both the hungry state [17] and in our fed state controlled for liking. This suggests that the right posterior STS activation may reflect a food’s biological relevance, irrespective of satiety and independent of food preference. However, to obtain more insight in the exact function of the STS in food choice and how this may be modulated by hunger and satiety, more research is needed.

Futhermore, we examined the differences between food and non-food choices. We found increased activation in the left insula, left superior temporal sulcus, left posterior cingulate gyrus and left (pre)cuneus, in response to food compared with non-food choices.

The insula is known for its involvement in value-based decision making. It integrates internal state and sensory signals and is important during response selection. In addition, it integrates information about the salience and relative value of stimuli [44]. Previous studies found significantly stronger activation in both the left insula and left posterior cingulate gyrus during food viewing tasks [18]. Both the insula [45–48] and the left posterior cingulate gyrus [49–52], were also found to be active during choice valuation tasks. Furthermore, activation in the posterior cingulate gyrus has been found to correlate with monetary reward magnitude [49–51] and willingness to pay for primary rewards (e.g. food) [52]. Although in our study the foods were most likely devaluated due to satiety, insula activation and posterior cingulate gyrus activation found in food viewing studies and choice valuation studies suggest that, despite a low motivation to eat, food items were more salient than non-food items. Other studies support this view and showed that non-food stimuli attract less attention than food images in both eye tracking [35,36] and neuroimaging studies using visual food cues [12,18].

During food choice, activation in the (pre)-cuneus and the left superior temporal sulcus was also increased. The precuneus is especially known for its involvement in attention [53,54]. This suggests that the increased activation in the precuneus reflects increased attention for the food pairs compared with the non-food pairs. The left STS is involved in simple moral decisions versus semantic decisions [55]. Increased activation in this region during food choice compared with non-food choice might reflect the different nature of the choices made, namely simple decisions between two office utensils versus more complex decisions between which food one would most want to eat.

In conclusion, we observed increased insula, posterior cingulate gyrus and precuneus activation during food choice versus non-food choice. This suggests that the food stimuli were more salient than the non-food stimuli despite the low motivation to eat. In addition, in line with our hypothesis, we did not find major differences between high versus low calorie choices between equally-liked food items in the absence of hunger. The right superior temporal sulcus was the only region found to be stronger activated during high calorie compared with low calorie choice independent of liking. Together with previous studies, this may suggest that right STS activation during food evaluation and choice reflects the food’s biological relevance independent of food preference. This novel finding warrants further research into the effects of hunger state and weight status on right STS, which may provide a marker of biological relevance.

## Supporting Information

S1 Fig Overlapping brain regions during food choice vs non-food choice, high calorie choice and low calorie choice. Shown are binary thresholded T-maps, superimposed on the mean anatomical image of all subjects. a: thresholded T-map during food vs non-food choice; b:thresholded T-map during high calorie choice; c: thresholded T-map during low calorie choice; d: All three thresholded T-maps. In purple overlap between FC-NFC and HCC, in yellow overlap between HCC and LCC and in white (indicated by the black circle) overlap in all three T-maps in the left insula at MNI (-33, 14, -10).
(TIF)

S1 PDF Overview of food stimuli. (PDF)

S1 Table Movement. (DOCX)

S2 Table Energy content of the test meal (Nutridrink). (DOCX)

S3 Table High and low calorie choice ratings per subject. (DOCX)

S4 Table Brain regions with stronger activation in response to low calorie food choice with stronger activation in response to low calorie food choices. (DOCX)

S5 Table Brain regions with stronger activation in response to high calorie food choice. (DOCX)
