# Supplementary figures and images for: Functional MRI of Challenging Food Choices: Forced Choice between Equally Liked High- and Low-Calorie Foods in the Absence of Hunger
Source: PLoS One. 2015 Jul 13;10(7):e0131727. doi: 10.1371/journal.pone.0131727 (PMC4500585; doi:10.1371/journal.pone.0131727)

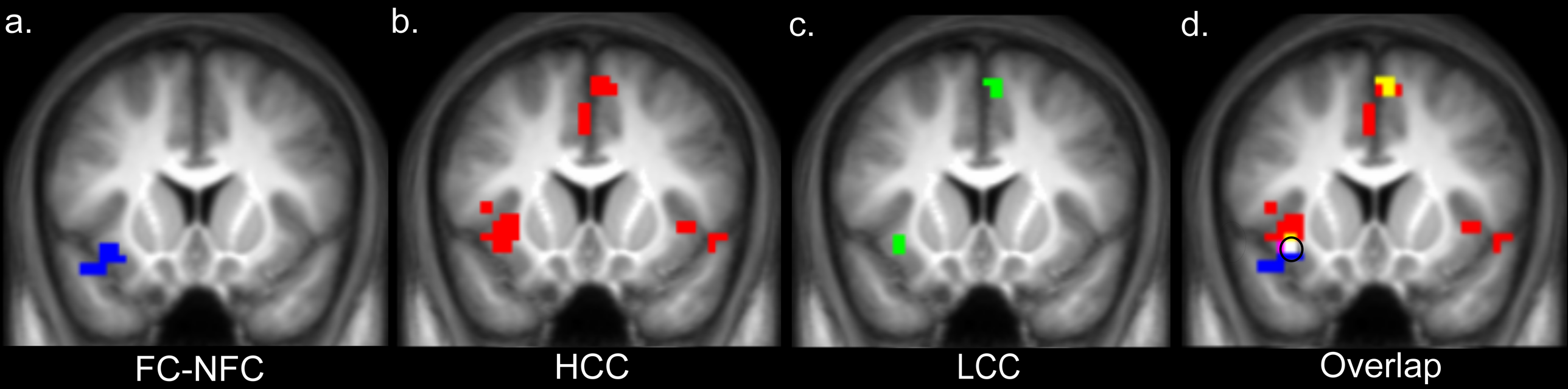

Supplement: S1 Fig — Shown are binary thresholded T-maps, superimposed on the mean anatomical image of all subjects. a: thresholded T-map during food vs non-food choice; b:thresholded T-map during high calorie choice; c: thresholded T-map during low calorie choice; d: All three thresholded T-maps. In purple overlap between FC-NFC and HCC, in yellow overlap between HCC and LCC and in white (indicated by the black circle) overlap in all three T-maps in the left insula at MNI (-33, 14, -10). (TIF) [file pone.0131727.s001.tif]
