# Supplementary material for: Functional MRI of Challenging Food Choices: Forced Choice between Equally Liked High- and Low-Calorie Foods in the Absence of Hunger
Source: PLoS One. 2015 Jul 13;10(7):e0131727. doi: 10.1371/journal.pone.0131727 (PMC4500585; doi:10.1371/journal.pone.0131727)
Supplement: S1 Table — (DOCX) [file pone.0131727.s003.docx]

|  | **Translation (mm)** | |  | **Rotation (◦)** | |
| --- | --- | --- | --- | --- | --- |
| **Coordinates** | **Mean ± SD** | **range** |  | **Mean ± SD** | **range** |
| **x** | 0.04 ± 0.14 | -0.42 - 0.48 |  | -0.0003 ± 0.004 | -0.0263 – 0.0397 |
| **y** | -0.44 ± 0.23 | -1.86 – 0.87 |  | -0.0006 ± 0.003 | -0.0142 – 0.0162 |
| **z** | 0.03 ± 0.27 | -1.63 – 1.18 |  | -0.0002 ± 0.002 | -0.0104 – 0.0063 |

**S1 Table. Movement.**
