# Supplementary material for: Functional MRI of Challenging Food Choices: Forced Choice between Equally Liked High- and Low-Calorie Foods in the Absence of Hunger
Source: PLoS One. 2015 Jul 13;10(7):e0131727. doi: 10.1371/journal.pone.0131727 (PMC4500585; doi:10.1371/journal.pone.0131727)
Supplement: S2 Table — (DOCX) [file pone.0131727.s004.docx]

| **Content of the protein drink:** | **Amount per 100 ml** |
| --- | --- |
| Energy | 150/625 kcal/kJ |
| Fats (amount saturated) | 5.8 g (0,6g) |
| Carbohydrates | 18.4 g |
| proteins | 5.9 g |

**S2 Table. Energy content of the test meal (Nutridrink).**
