# Supplementary material for: Functional MRI of Challenging Food Choices: Forced Choice between Equally Liked High- and Low-Calorie Foods in the Absence of Hunger
Source: PLoS One. 2015 Jul 13;10(7):e0131727. doi: 10.1371/journal.pone.0131727 (PMC4500585; doi:10.1371/journal.pone.0131727)
Supplement: S3 Table — (DOCX) [file pone.0131727.s005.docx]

|  | **High calorie choices** | | | | |  | **Low calorie choices** | | | | |
| --- | --- | --- | --- | --- | --- | --- | --- | --- | --- | --- | --- |
| ID | No. of choices | Actual cal. ^1^  (mean± $SD$) | Perceived^2^ cal. (mean± $SD$) | Liking^2^  (mean±$SD$) | Health^2^  (mean±SD) |  | No. of choices | Actual cal. ^1^  (mean±SD) | Perceived^2^ cal. (mean± $SD$) | Liking^2^  (mean± $SD$) | Health^2^  (mean± $SD$) |
| 1 | 20 | 397.9 ± 97.5 | 7.6 ± 1.1 | 7.4 ± 1.3 | 3.1 ± 1.6 |  | 24 | 136.7 ± 142.2 | 2.5 ± 1.4 | 6.9 ± 1.5 | 7.3 ± 2.2 |
| 2 | 12 | 389.9 ± 149.6 | 6.5 ± 1.3 | 6.8 ± 1.8 | 3 ± 1.5 |  | 31 | 154.8 ± 155.2 | 2.2 ± 1.2 | 7.8 ± 1.3 | 7.7 ± 1.5 |
| 3 | 15 | 318.1 ± 123.8 | 7.2 ± 1.2 | 6.9 ± 0.9 | 3 ± 1.5 |  | 21 | 74.6 ± 76.9 | 3.5 ± 1.1 | 7 ± 0.8 | 6.8 ± 0.9 |
| 4 | 20 | 332.3 ± 124.9 | 7.5 ± 1.1 | 5.6 ± 2.3 | 2.5 ± 1.5 |  | 20 | 205.8 ± 150.2 | 3.2 ± 1.5 | 6.3 ± 1.9 | 7.1 ± 1.8 |
| 5 | 15 | 333.7 ± 145.1 | 7.3 ± 1 | 7.8 ± 1 | 4.2 ± 2.2 |  | 24 | 146.5 ± 138.7 | 4.3 ± 1.1 | 7.5 ± 1.2 | 6.7 ± 1.5 |
| 6 | 13 | 318.9 ± 127.8 | 6.3 ± 0.8 | 7.5 ± 0.5 | 4.2 ± 1.4 |  | 26 | 92.2 ± 95.5 | 2.6 ± 0.9 | 7.5 ± 0.6 | 7.2 ± 0.9 |
| 7 | 13 | 367.6 ± 147.8 | 7.5 ± 0.9 | 5.7 ± 2.6 | 3.9 ± 1.8 |  | 28 | 202.5 ± 173.5 | 3.3 ± 1.6 | 5.3 ± 2.2 | 6.7 ± 1.6 |
| 8 | 19 | 340.2 ± 114.2 | 7.4 ± 1.2 | 6.6 ± 1.9 | 2.6 ± 1.4 |  | 21 | 145.3 ± 171.4 | 2.8 ± 1.7 | 6.5 ± 1.6 | 6.8 ± 1.8 |
| 9 | 15 | 351.7 ± 123.6 | 7.9 ± 1.1 | 6.5 ± 2.8 | 2.9 ± 1.8 |  | 13 | 155.2 ± 180.5 | 3.9 ± 1 | 6.4 ± 2.7 | 7.1 ± 1.4 |
| 10 | 19 | 416.6 ± 109.7 | 7.8 ± 1.2 | 6.6 ± 1.9 | 3.4 ± 2.3 |  | 20 | 161.7 ± 168.4 | 3.1 ± 1.6 | 6.8 ± 1.8 | 7.5 ± 2.1 |
| 11 | 18 | 384.5 ± 121.4 | 8.3 ± 0.8 | 7.6 ± 1.3 | 2.6 ± 2.3 |  | 22 | 163.8 ± 134.8 | 4.5 ± 1.9 | 7.3 ± 2.4 | 7 ± 2.7 |
| 12 | 19 | 352.6 ± 137 | 7.2 ± 1.3 | 6.3 ± 1.3 | 2.6 ± 1.3 |  | 26 | 226 ± 165.4 | 2.2 ± 1.1 | 5.8 ± 1.3 | 6.5 ± 1.9 |
| 13 | 26 | 386.3 ± 97.2 | 8.1 ± 1 | 6 ± 2.4 | 2.3 ± 1.8 |  | 18 | 140.4 ± 118.6 | 4.1 ± 1.6 | 6.2 ± 2.9 | 7.7 ± 1.2 |
| 14 | 20 | 310.1 ± 105.3 | 7.2 ± 1.1 | 6.4 ± 1.6 | 2.8 ± 1 |  | 24 | 168.2 ± 170.1 | 3 ± 1.6 | 6.3 ± 1.4 | 6.8 ± 1.7 |
| 15 | 21 | 375 ± 124.6 | 7.5 ± 1.8 | 7.4 ± 2.3 | 1.9 ± 1.3 |  | 22 | 171.5 ± 176.9 | 2.7 ± 1.4 | 5.9 ± 2.9 | 7.1 ± 2.6 |
| 16 | 11 | 409 ± 106.6 | 7.3 ± 1.7 | 6.6 ± 2.8 | 3.2 ± 2.5 |  | 38 | 185.1 ± 160.4 | 2.4 ± 1.7 | 7.6 ± 2 | 7.2 ± 2.2 |
| 17 | 11 | 406.4 ± 69 | 7.5 ± 1.2 | 6.6 ± 1.2 | 3.8 ± 1.3 |  | 27 | 132.9 ± 142.8 | 2.9 ± 1.5 | 7.4 ± 0.8 | 7 ± 1.2 |
| 18 | 18 | 408.4 ± 84.4 | 8.1 ± 1.4 | 6.7 ± 2.5 | 2.9 ± 2 |  | 29 | 126.6 ± 138.6 | 2.6 ± 1.5 | 6.9 ± 2.5 | 7.9 ± 2.2 |
| 19 | 23 | 363.3 ± 128.2 | 6.7 ± 1.1 | 5.7 ± 1.8 | 4.5 ± 1.5 |  | 11 | 154.2 ± 158.3 | 4.1 ± 1.5 | 6.8 ± 1.9 | 7.2 ± 2.1 |

**S3 Table. High and low calorie choice ratings per subject**

^1^ Actual caloric content kcal per 100 grams; ^2^ 9-point Likert scale.
