# Supplementary material for: Functional MRI of Challenging Food Choices: Forced Choice between Equally Liked High- and Low-Calorie Foods in the Absence of Hunger
Source: PLoS One. 2015 Jul 13;10(7):e0131727. doi: 10.1371/journal.pone.0131727 (PMC4500585; doi:10.1371/journal.pone.0131727)
Supplement: S4 Table — (DOCX) [file pone.0131727.s006.docx]

**S4 Table. Brain regions with stronger activation in response to low calorie food choice**

|  |  | **Peak MNI-coordinates (mm)** | | | |  |
| --- | --- | --- | --- | --- | --- | --- |
| **Region** | **k** | **x** | **y** | **z** | **T** | **Z** |
| R, Cerebellum | 276 | 30 | -60 | -26 | 11.53 | 6.12 |
| R, Inferior Occipital lobe |  | 30 | -84 | -2 | 10.64 | 5.91 |
| R, Cerebellum |  | 34 | -44 | -30 | 7.72 | 5.07 |
| L, Cerebellum | 170 | -34 | -64 | -22 | 10.73 | 5.93 |
| L, Inferior occipital lobe |  | -22 | -88 | -10 | 7.65 | 5.04 |
| L, Cerebellum |  | -34 | -52 | -26 | 7.60 | 5.02 |
| R, Middle cingulate gyrus | 82 | 2 | 20 | 38 | 9.42 | 5.59 |
| L, Supplemental motor area |  | -2 | 20 | 50 | 8.56 | 5.34 |
| R, Supplemental motor area |  | 6 | 12 | 58 | 6.65 | 4.67 |
| R, Insula | 31 | 42 | 24 | -6 | 9.13 | 5.51 |
| L, Middle occipital lobe | 25 | -34 | -76 | 2 | 8.84 | 5.43 |
| L, Supramarginal gyrus | 95 | -58 | -28 | 34 | 8.59 | 5.35 |
| L, Inferior parietal lobe |  | -42 | -48 | 46 | 7.86 | 5.11 |
| L, Postcentralgyrus |  | -50 | -24 | 54 | 7.83 | 5.10 |
| L, Insula | 33 | -30 | 24 | -6 | 8.28 | 5.25 |
| L, Inferior frontal gyrus | 14 | -46 | 36 | 10 | 8.10 | 5.19 |
| L, Midbrain | 25 | -6 | -28 | -18 | 8.06 | 5.18 |
| L, Thalamus |  | -2 | -20 | -2 | 7.49 | 4.98 |
| R, Midbrain |  | 2 | -24 | -22 | 6.89 | 4.76 |

*Peaks are reported for all clusters ≥ 10 voxels at p<0.05 FWE corrected for multiple comparisons; L = left and R= right hemisphere.*
