# Supplementary material for: Functional MRI of Challenging Food Choices: Forced Choice between Equally Liked High- and Low-Calorie Foods in the Absence of Hunger
Source: PLoS One. 2015 Jul 13;10(7):e0131727. doi: 10.1371/journal.pone.0131727 (PMC4500585; doi:10.1371/journal.pone.0131727)
Supplement: S5 Table — (DOCX) [file pone.0131727.s007.docx]

**S5 Table. Brain regions with stronger activation in response to high calorie food choice.**

|  |  | **Peak MNI-coordinates (mm)** | | |  |  |
| --- | --- | --- | --- | --- | --- | --- |
| **Region** | **k** | **x** | **y** | **z** | **T** | **Z** |
| R, Inferior Occipital lobe | 415 | 30 | -84 | -2 | 13.79 | 6.57 |
| R, Cerebelum |  | 30 | -56 | -26 | 11.36 | 6.08 |
| R, Inferior Occipital lobe |  | 30 | -84 | -18 | 10.27 | 5.82 |
| R, Middle cingulate gyrus | 115 | 6 | 20 | 34 | 11.34 | 6.07 |
| L, Supplemental motor area |  | -2 | 24 | 46 | 9.11 | 5.51 |
| R, Supplemental motor area |  | 6 | 12 | 58 | 7.16 | 4.87 |
| L, Cerebellum | 341 | -34 | -64 | -18 | 11.09 | 6.02 |
| L, Inferior occipital lobe |  | -26 | -88 | -10 | 8.83 | 5.42 |
| L, Middle occipital lobe |  | -34 | -76 | 2 | 8.71 | 5.39 |
| L, Postcentralgyrus | 198 | -46 | -28 | 58 | 10.92 | 5.98 |
| L, Supramarginalgyrus |  | -62 | -24 | 26 | 10.03 | 5.76 |
| L, Supramarginalgyrus |  | -58 | -28 | 38 | 9.51 | 5.62 |
| L, Insula | 64 | -34 | 12 | -6 | 10.66 | 5.92 |
| L, Midbrain | 46 | -10 | -28 | -14 | 9.68 | 5.67 |
| R, Midbrain |  | 10 | -24 | -18 | 7.66 | 5.05 |
| R, Midbrain |  | -2 | -20 | -18 | 7.19 | 4.88 |
| L, Cuneus | 31 | -18 | -72 | 34 | 8.31 | 5.26 |
| L, Inferior parietal lobe |  | -26 | -68 | 42 | 6.92 | 4.77 |
| L, Superior parietal lobe |  | -30 | -60 | 54 | 6.74 | 4.70 |
| R, Insula | 41 | 34 | 20 | 6 | 8.07 | 5.19 |
| R, Insula |  | 42 | 16 | -2 | 7.84 | 5.11 |
| R, Superior temporal sulcus |  | 50 | 16 | -10 | 7.48 | 4.98 |
| L, Precentral gyrus | 13 | -46 | 4 | 22 | 7.38 | 4.95 |

*Peaks are reported for all clusters ≥ 10 voxels at p<0.05 FWE corrected for multiple comparisons; L = left and R= right hemisphere.*
